# Predictivity of the Prognostic Nutritional Index and Systemic Inflammation Index for All-Cause In-Hospital Mortality in Geriatric and Adult COVID-19 Inpatients

**DOI:** 10.3390/jcm13154466

**Published:** 2024-07-30

**Authors:** Sibel Cavdar, Sumru Savas, Sezai Tasbakan, Abdullah Sayıner, Ozen Basoglu, Pervin Korkmaz, Fehmi Akcicek

**Affiliations:** 1 Division of Geriatrics, Department of Internal Medicine, İzmir City Hospital, 35540 İzmir, Türkiye; 2 Division of Geriatrics, Department of Internal Medicine, Ege University Hospital, 35100 İzmir, Türkiye; emine.sumru.savas@ege.edu.tr (S.S.); fehmi.akcicek@gmail.com (F.A.); 3 Department of Respiratory Medicine, Ege University Hospital, 35100 İzmir, Türkiye; sezai72000@yahoo.com (S.T.); sayiner2011@gmail.com (A.S.); ozenbasoglu@yahoo.com (O.B.); 4 Department of Respiratory Medicine, Medicana İstanbul International Hospital, 34520 İstanbul, Türkiye; pervinkorkmaz@yahoo.com

**Keywords:** COVID-19, aged, geriatric, prognostic nutritional index, systemic immune inflammation index

## Abstract

**Background:** The prognostic nutritional index (PNI) and the systemic immune inflammation index (SII) have been used as simple risk-stratification predictors for COVID-19 severity and mortality in the general population. However, the associations between these indices and mortality might differ due to age-related changes such as inflammaging and several comorbid conditions in older patients. Therefore, we aimed to compare the predictivity of the PNI and SII for mortality among hospitalized older patients and patients under 65 years old. **Methods:** Patients hospitalized with COVID-19 from March 2020 to December 2020 were retrospectively included. The PNI and SII were calculated from hospital records within the first 48 h after admission. Data were evaluated in the whole group and according to age groups (≥65 < years). Receiver operating characteristic curves were drawn to evaluate the predictivity of the PNI and SII. **Results:** Out of 407 patients included in this study, 48.4% (n = 197) were older patients, and 51.6% (n = 210) were under 65 years old. For mortality, the area under the curve (AUC) of the PNI and SII in the adult group (<65 years) was 0.706 (95% CI 0.583–0.828) (*p* = 0.003) and 0.697 (95% CI 0.567–0.827) (*p* < 0.005), respectively. The AUC of the PNI and SII in the older group was 0.515 (95% CI 0.427–0.604) (*p* = 0.739) and 0.500 (95% CI 0.411–0.590) (*p* = 0.993). **Conclusions:** The accuracy of the PNI and SII in predicting mortality in adult COVID-19 patients seemed to be fair, but no association was found in geriatric patients in this study. The predictivity of the PNI and SII for mortality varies according to age groups.

## 1. Introduction

Severe acute respiratory syndrome coronavirus 2 (SARS-CoV-2) infection has a more severe course and a higher mortality in older patients [1]. More than 90% of deaths from Coronavirus disease 2019 (COVID-19) occur in patients aged 60 years and over [2]. Older individuals are the most vulnerable group to COVID-19 infection due to several factors such as susceptibility to malnutrition and an increased inflammatory status because of comorbidities, inflammaging, and immunosenescence [2,3,4]. Moreover, COVID-19 is known as a multisystem inflammatory disease with the involvement of proinflammatory cytokines [5]. Inflammatory responses play a fundamental role in COVID-19-related mortality [6].

Various numbers of risk factors have been identified to have a potential impact on increasing the morbidity and mortality of COVID-19 in adults, including old age, male sex, pre-existing comorbidities, and changes in laboratory indices and proinflammatory cytokines, as well [1,2]. There are various inflammatory markers, such as the neutrophil/lymphocyte ratio (NLR), derived NLR (d-NLR), platelet/lymphocyte ratio (PLR), lymphocyte/monocyte ratio (LMR), C-reactive protein/lymphocyte ratio (CLR), systemic immune inflammation index (SII), C-reactive protein (CRP)-to-albumin ratio (CAR), high-sensitivity CAR (hsCAR), CRP-to-prealbumin ratio (CPAR), aggregate index of systemic inflammation (AISI), and prognostic nutritional index (PNI), for risk-stratifying and prognosticating COVID-19 severity and mortality [6,7]. In a retrospective study with 450 patients aged between 38 and 78, PNI was superior to NLR in predicting mortality, and CAR was superior to NLR in predicting disease severity in another retrospective study with 90 patients [6,8,9]. In a retrospective study with 119 patients, SII was superior to NLR, d-NLR, MLR, AISI, and PLR in predicting mortality [6,7,10]. According to a review by Karimi et al., SII, PNI, CAR, and hsCAR might produce more promising results than NLR, and PLR seems to lack the potential to predict mortality [6]. LMR might have limited benefits in prognosticating COVID-19, and its abilities seem to be lower than those of NLR and PLR [6,11,12,13]. Since the PNI and SII were found to be promising indexes for predicting COVID-19 mortality, we focus on these two indexes.

The prognostic nutritional index is an objective and simple assessment index that reflects the immune/inflammatory–nutritional status, calculated using the serum albumin level and lymphocyte count [14]. It is easy to implement and it is used as a prognostic marker in various clinical settings, such as cancer, stroke, and cardiovascular diseases [15]. In a meta-analysis, it was shown that the PNI is a predictor for COVID-19 severity and mortality in hospitalized patients [16]. In another systematic review and meta-analysis by Hung et al., it was demonstrated that there is a negative association between the PNI and COVID-19 mortality [17]. A similar index which reflects the inflammatory and immune status of patients is the systemic immune inflammation index, which includes platelet, neutrophil, and lymphocyte counts [7,18,19]. The SII has been used as a predictive marker in a variety of tumors and inflammatory diseases [20,21]. The SII has also been shown to be an independent and powerful predictor of worse clinical outcomes and in-hospital mortality in COVID-19 patients [5,7]. In a recent literature review by Kosidlo et al., the SII was reported to be associated with mortality, although there are a few studies with contradictory findings [22].

Although the PNI and SII have taken their place in the literature as predictive indexes for COVID-19 mortality, their importance according to age groups and in older people is not clear. Some physiological and non-physiological alterations that occur in older adults may change the predictivity of these indices.

In older individuals, the balance between the proinflammatory and anti-inflammatory response is impaired. Increased amounts of proinflammatory cytokines in the plasma of an older individual without an overt manifestation of inflammatory disease have broad implications for a proinflammatory and immunosuppressive state [23]. The shift to an excessive increase in the proinflammatory response might cause a cytokine storm in the older population [24]. Due to the contribution of several factors, a cytokine storm may be fatal in some older patients [24]. It is also known that hyperinflammation plays an important role in viral pathogenesis [6]. Since the PNI and SII indices show the immune inflammatory and nutritional status of patients, the presentation of those conditions might be different in patients under versus over 65 years of age. Additionally, the prognostic significances might also differ. Though there are emerging studies investigating the relationship between prognostic indices, such as the PNI and SII, and COVID-19 mortality and severity in the general population, studies on geriatric patient groups are scarce. To our knowledge, there is only one study which evaluated the predictivity of the SII in the older and adult patients comparatively [25], and there are no studies investigating the predictivity of the PNI or that of the PNI concomitantly with the SII in the older population in comparison with the adult group for COVID-19 mortality. Therefore, we aimed to investigate the relationship between the PNI and SII and COVID-19 mortality in hospitalized patients and to compare their predictive value in older and adult-aged (<65 years of age) groups to show whether these indices can be used in the older patient group as in adult patients, despite many changes in the older population that may affect these indices. Though our aim was to compare adult and older patients with COVID-19, we also evaluated the whole group in order to make a comparison with the literature because studies according to age groups are scarce.

## 2. Materials and Methods

### 2.1. Subjects

Patients over 18 years of age hospitalized for COVID-19 infection diagnosis at Ege University Respiratory Medicine Department from 20 March 2020 to 21 December 2020 were included in this study. We conducted a retrospective study on patients with confirmed or clinically suspected COVID-19 who met one of the following criteria: (1) patients with a real-time reverse-transcriptase polymerase chain reaction (PCR) test of an oropharyngeal swab which was positive for SARS-CoV-2 or (2) patients who were highly suspected to have COVID-19 based on the World Health Organization’s interim guidance, comprising those who had a history coherent with COVID-19 and had predominantly peripheral ground-glass opacities accompanied by consolidation on chest computed tomography or ground-glass opacity alone, not perfectly elucidated by nodules, lobar collapse, or volume overload [16]. The exclusion criteria were pregnancy and missing data at admission. Patients taken directly to the intensive care unit (ICU) were also excluded. Patients admitted to the ward with no or partial support were included. The patients were evaluated as the geriatric (older) (≥65 years of age) and adult (<65 years of age) groups and as the whole population.

### 2.2. Measures

The data of all patients were obtained until in-hospital death or discharge from the hospital. The socio-demographic information and clinical and laboratory characteristics of the patients were retrieved from electronic medical records. Data on age, sex, comorbidities, pulse and respiratory rates, need for intensive care unit hospitalization, length of hospital stay, and outcomes (discharge or death), as well as laboratory data within the first 48 h after admission, were noted. 

Laboratory parameters included complete blood count (leukocytes, lymphocytes, neutrophils, and platelets), NLR, urea, creatinine, lactate dehydrogenase (LDH), albumin, CRP, ferritin, and procalcitonin.

The PNI was calculated according to the following formula: PNI = 10 × serum albumin (g/dL) + 0.005 × peripheral lymphocyte count (/mm^3^) [26]. The SII was calculated according to the following formula: SII = neutrophil count × platelet count/lymphocyte count [7].

### 2.3. Statistical Analyses

The normal distribution of the variables was examined using analytical methods (Kolmogorov–Smirnov or Shapiro–Wilk tests depending on the number of the participants in the groups). Descriptive analyses were given as means ± standard deviations (SDs) for normally distributed variables and medians (minimum–maximum) for variables without normal distribution. Categorical variables were given as frequencies and percentages. For numerical data, the *t*-test was used for independent groups with normal distribution and the Mann–Whitney U test for non-normally distributed variables. Pearson’s chi squared x^2^ or Fisher’s exact test were used in the analysis of categorical data. Receiver operating characteristic (ROC) analysis was used to determine the predictivity of the markers. The effects of the PNI and SII indices on predicting mortality were analyzed with ROC curve analyses with an area under curve (AUC). The AUC is a measure of the overall diagnostic accuracy of a test. An AUC > 0.8 indicates good, 0.6–0.8 fair, and <0.6 indicates poor diagnostic accuracy [27]. *p*-values < 0.05 were considered statistically significant. Statistical analyses were performed using IBM^®^ SPSS^®^ 22 (SPSS Inc., Chicago, IL, USA) software.

## 3. Results

The data of 428 patients were retrospectively evaluated. One patient was pregnant, and twenty patients with missing data were excluded. Finally, a total of 407 patients were enrolled in this study. There were 197 patients (48.4%) in the geriatric group (82 females and 115 males), and 210 patients (51.6%) were in the group under 65 years of age (87 females and 123 males). The median age was 64 (18–95) years in the general population, 52 (18–64) years in the adult group, and 74 (65–95) in older patients. The characteristics of the total group according to survival status are shown in Supplementary Table S1.

In the whole population categorized according to mortality, deceased patients were significantly older than the non-deceased patients (74 vs. 62, *p* < 0.001), while there was no significant difference in terms of gender (*p* = 0.387). The PNI was lower (41.6 vs. 43.9, *p* < 0.001) and the SII was higher (1179 vs. 816, *p* = 0.013) among non-survivors in the whole group. The length of hospital stay and intensive care unit transfer were significantly higher in deceased patients (Supplementary Table S1).

According to age groups, comorbidities were reported significantly more often in older patients (56.4% vs. 43.6%, *p* < 0.001). The frequencies of congestive heart failure, coronary artery disease, hypertension, diabetes mellitus, cerebrovascular disease, and chronic obstructive pulmonary disease, as well as the use of medications, were significantly higher in the older group (*p* = 0.035, *p* = 0.001, *p* < 0.001, *p* = 0.001, *p* < 0.001, *p* = 0.002, *p* = 0.001 respectively). Immunosuppressive medication use was significantly higher in the adult group than in the older patients (15.2% vs. 8.7%, *p* = 0.044). Of the geriatric patients, 46.9% were transferred to the ICU as compared to 28% of non-geriatric adult patients (*p* < 0.001). Of all, 29.1% of geriatric patients, and 10% of adult patients died (*p* < 0.001). Compared with the adult group, older patients had lower lymphocyte counts (1109 vs. 1628, *p* < 0.005), lower albumin (35.5 vs. 38.6, *p* < 0.001), and PNI (41.15 vs. 47.68, *p* < 0.001). Neutrophil/lymphocyte ratios (7.36 vs. 5.23, *p* < 0.001), SII (1746 vs. 1234, *p* = 0.003), urea (56.7 vs. 35.7, *p* < 0.001), and procalcitonin levels (0.76 vs. 0.29, *p* < 0.001) were higher in the geriatric group.

The general characteristics of the older population and the adult group are shown in Table 1 according to survival status (Table 1). The laboratory values of the older population and the adult group according to survival status are given in Table 2.

### Predictive Accuracy of Inflammation Indices According to Age Groups

**Figure 1:**
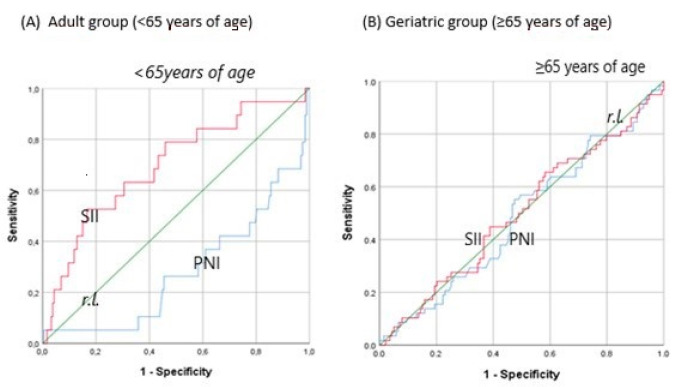
ROC curves of the PNI and SII in the adult group and geriatric group for mortality. SII: systemic inflammation index, PNI: prognostic nutritional index, r.l.: reference line.

The area under the curve of the PNI and SII in the adult group was 0.706 (95% CI 0.583–0.828) (*p* = 0.003) and 0.697 (95% CI 0.567–0.827) (*p* < 0.005), respectively. The AUC of the PNI and SII in the geriatric group was 0.515 (95% CI 0.427–0.604) (*p* = 0.739) and 0.500 (95% CI 0.411–0.590), respectively (*p* = 0.993). In the total population, the AUC of the PNI was 0.631 (95% CI 0.564–0.698) (*p* < 0.001) and the AUC of the SII was 0.591 (95% CI 0.519–0.662) (*p* = 0.013). The ROC curves of the PNI and SII in the adult group and geriatric group for mortality are shown in Figure 1 (Figure 1), while the ROC curves of the PNI and SII of the whole group are shown in the Supplementary Materials.

## 4. Discussion

In this study, we found that while in patients under the age of 65, the PNI and SII can distinguish between COVID-19 survivors and non-survivors, the two indices do not have a predictive power in the group aged 65 and over.

Advanced age, higher rates of ICU transfer, and longer hospital stay, as well as higher levels of procalcitonin, ferritin, LDH, urea, and creatinine, were significantly different for non-survivors in both the adult and geriatric group. All these factors are defined as independent predictors of COVID-19 in-hospital mortality in different studies [28,29,30]. Comorbidities which are typical for older individuals but can befall younger individuals, including cardiovascular disease, type 2 diabetes mellitus, chronic respiratory disease, chronic kidney disease, arterial hypertension, and various malignancies, significantly increase the death rate of COVID-19 patients compared to those with no pre-existing conditions [23,31]. In our study, though older patients had more comorbidities than adults, there was not a difference between the survivor and non-survivor group of older patients in terms of comorbidities. Geriatric non-survivors only had significantly more chronic renal failure. In a study conducted in older patients in which the primary endpoint was all-cause in-hospital mortality, chronic kidney disease and dementia were found to be the only independent risk factors of mortality among other comorbidities [32]. Chronic renal disease is associated with an accelerated aging process, which leads to alterations in the immune system [33]. Aging is also associated with reduced kidney function and it provides an overall environment facilitating a stronger inflammasome activation in COVID-19 patients [23]. Among patients over 80 years old hospitalized with COVID-19, not only a worse clinical and radiological presentation of the disease, but also increased age, dementia, and impairment in activities of daily living were strong risk factors for in-hospital death, regardless of disease severity [34].

Patients with COVID-19 usually have increased levels of serologic indicators of inflammation, such as CRP, LDH, erythrocyte sedimentation rate, and procalcitonin [6]. In our study, procalcitonin, ferritin, and LDH were significantly high in the deceased group of both adults and older patients. CRP was significantly higher in the deceased adult population. CRP may not determine mortality in older COVID-19 patients, especially due to age-related rearrangements in immunity and cytokine production [35]. However, in the general group of non-survivors, all inflammation indicators—CRP, LDH, procalcitonin, and ferritin—were significantly higher than in survivors, which is consistent with studies in the literature [36,37].

Various predictive factors have been studied to quickly identify patients who will deteriorate and need closer medical attention in the emergency room, ward, or intensive care unit for COVID-19 [14]. Different inflammatory markers such as NLR, CAR, MLR, PLR, albumin/globulin ratio, and the PNI and SII were evaluated in many studies in terms of their use for COVID-19 risk stratification [6,38]. Although NLR is the most evaluated inflammatory index in COVID-19 patients and seems to be a robust one, according to some study results, the PNI and SII were found to be superior to NLR [6,38]. The PNI and SII are two indices which can be used quickly and which have been shown to predict the severity and mortality of COVID-19 patients in several studies in the general population, usually regardless of age. In the literature about COVID-19, studies on these two indices focusing on older patients are scarce. There are some studies which aimed to describe the clinical characteristics of older inpatients with COVID-19 and to identify the risk factors for in-hospital mortality [32,39]. There are almost no studies investigating the predictivity of markers in geriatric and adult COVID-19 patients [25,40]. However, the predictivity of the PNI and SII might differ in these two groups due to age-related changes, especially in the hematologic [41] and immune systems [23,24]. In addition, there may be a decrease in plasma albumin levels in association with the degree of inflammation and poor nutritional status [42]. Finally, in the geriatric population, several comorbid conditions, such as diabetes mellitus, rheumatological diseases, chronic kidney disease, and other malignancies, may be inflammatory in nature and may reduce the usefulness of some markers [6]. Choosing the right marker for the right population to detect patients with serious health outcomes and poor prognosis is essential.

In terms of the PNI and SII, non-survivors in the general group had significantly lower PNI and higher SII scores than survivors in our study. When we evaluated the predictivity of the PNI and SII with ROC curves in the general group, the AUC values showed both the PNI and the SII might predict mortality; the PNI had a higher AUC value. All these findings are consistent with the previous reports for the PNI [17,38,43,44] and also the SII [22,45,46]. However, there are studies in the literature that do not fully support this finding, especially for the SII [18,38,47,48,49,50,51,52].

In the adult group, non-survivors had significantly lower PNI and higher SII scores than survivors, as in the general population, but the AUC values for both the PNI and SII were higher in adults than in the general population. In our study, the significance of these indices for the whole group in terms of ROC curves comes from the adult group, and it should be noted that the AUC of the whole group is lower than that of the adult group due to the contribution of the geriatric group’s non-significance. In the older group, there were no differences between survivors and non-survivors in terms of PNI and SII scores for in-hospital mortality. Moreover, according to the ROC curves, the PNI and SII did not predict mortality in older patients. In a review by Karimi et al., the need for future research on specific subgroups was emphasized to find out the most suitable biomarkers for different groups because biomarkers for different subgroups might differ from the overall population [6]. Studies comparing inflammatory markers in different age groups in COVID-19 patients are scarce [25,40]. Hassan et al. aimed to investigate the role of systemic inflammatory markers in predicting mortality in adult and older COVID-19 patients. In non-survivor adult and older patients, SII scores were significantly higher than among survivor patients. On the other hand, a multivariate Cox regression model showed that white blood cell (WBC) and neutrophil counts, d-NLR, and the SII in adult patients and WBC count and neutrophil count in older patients were significantly associated with survival. As a result, the SII was not found to be associated with survival in older patients [25]. Although most developed scores include age among the factors evaluated for risk prediction, none of these tools have been validated in a geriatric population [53]. A study by Pelegatti et al. evaluated the prognostic stratification ability of the 4C Mortality Score in different age groups for COVID-19. The ability of this score to identify patients at a higher risk of in-hospital mortality was similar in different age groups [41]. The 4C Mortality Score consists of eight variables: age, sex, respiratory rate, oxygen saturation, number of comorbidities, level of consciousness, blood urea nitrogen, and CRP [54]. This score may be a valid predictor in all age groups, possibly because it includes both clinical and laboratory parameters. The SII and PNI, which only include inflammatory markers, can be affected by age for a variety of reasons and through numerous mechanisms. Older patients tend to develop a cytokine storm and its consequences more frequently than younger people because of mitochondrial dysfunction, increased oxidative stress, comorbidities, which are mostly chronic inflammatory diseases, decreased output of steroid hormones, poor nutrition, and restricted activity, as well as immune dysregulation, immunosenescence and inflammaging [23]. In our study, compared with the adult group, geriatric patients had significantly lower lymphocyte count, albumin, and PNI levels. Neutrophil/lymphocyte ratios and SII scores were significantly higher in the geriatric group. However, despite this significant difference, none of these parameters could distinguish between deceased and non-deceased people in older patients.

## 5. Conclusions

In this study, the predictivity of the PNI and SII for all-cause in-hospital mortality in geriatric and adult COVID-19 patients was comparatively evaluated. To our knowledge, this is the first study to examine the prognostic significance of the PNI and SII on mortality together according to age groups (geriatric and adult). Stratifying by age group is important because of the physiological and non-physiological changes in the older population which may affect the significance of inflammatory indices while predicting mortality and prognosis. These may lead to differences when prognosticating patients in clinical practice. So, though these indices seem to be promising for predicting COVID-19 mortality in the literature, they had previously been evaluated in the general population regardless of age group. In our study, when the whole population was examined without stratifying by age, like in the literature, the PNI and SII were able to predict mortality with fair and poor AUC values, respectively. In the literature, compared with our study’s results in the general patient population, there are similar, higher, and lower AUC values for these indices. Though various studies have different AUC significance values, most of them indicate that these indices can predict COVID-19 mortality for the whole patient group, except for a few studies using the SII. We hypothesize that, if the studies in the literature were divided into older and adult age groups, these indices might not be predictive for older patients. The results of this study show that while the PNI and SII predicted COVID-19 mortality in the adult group, they did not predict it in geriatric patients. Thus, the PNI and SII can be used to evaluate prognoses in adult COVID-19 patients. While the PNI and SII are promising predictive indices for COVID-19, it should be remembered that the predictability of the PNI and SII for mortality may vary according to age groups, and for the older population, the importance of those indices need to be further researched in longitudinal studies. 

## 6. Limitations and Strengths

This is the first study to show the prognostic value of the PNI and SII together in COVID-19 patients according to age groups. The associations and predictivity of these inflammatory markers may vary according to age. The main strength of our study is that it has shown these indices are valid predictors of mortality in the non-geriatric adult group only. The main limitation of this study was that it was conducted in a single tertiary care center and that the data were retrospectively obtained from a registry. Data such as ECOG performance score or body mass index were not present in the data recorded during the COVID-19 period. So, those data could not be obtained due to this study’s retrospective design. Additionally, if other indices, such as CAR, NLR, or PLR, had also been examined, inflammatory indices in COVID-19 could have been evaluated with a broader perspective on the basis of age. Laboratory values were evaluated within 48 h of admission. If corticosteroid treatment had been added to the patients’ treatment during these two days, this may have changed their hemogram parameters, which are an important part in inflammatory indices. Not examining the treatments together with the laboratory parameters might be an important limitation.

## Figures and Tables

**Table 1:** Characteristics of the older population and the adult group according to survival status.

Variables	<65 Years	<65 Years		≥65 Years	≥65 Years	
	Survivors (n = 190)	Non-Survivors (n = 20)	*p*	Survivors (n = 139)	Non-Survivors (n = 58)	*p*
**Age, y**	51 (18–64)	59.5 (34–64)	**0.001**	72 (65–91)	78.5 (65–95)	**<0.001**
**Male, N (%)**	109 (57.4)	14 (70)	0.275	80 (57.6)	35 (60.3)	0.717
**Female, N (%)**	81 (42.6)	6 (30)	0.275	59 (42.4)	23 (39.7)	0.717
**Respiratory rate, min^−1^**	20 (14–40)	20 (18–32)	0.058	20 (16–36)	20.5 (16–30)	**0.002**
**Heart rate, bpm**	88.5 (37–149)	86.5 (60–128)	0.995	86 (55–142)	88 (63–141)	0.106
**Comorbidity, N (%)**	115 (60.5)	17 (85)	**0.031**	120 (87)	53 (91.4)	0.380
**CHF, N (%)**	3 (1.6)	2 (10)	**0.019**	10 (7.2)	3 (5.3)	0.759
**CAD, N (%)**	15 (7.9)	1 (5)	1.000	22 (15.9)	14 (24.6)	0.158
**HT, N (%)**	43 (22.6)	5 (25)	0.783	76 (55.1)	35 (61.4)	0.417
**DM, N (%)**	36 (18.9)	4 (20)	1.000	50 (36.2)	16 (28.1)	0.273
**CVD, N (%)**	1 (0.5)	0 (0)	0.745	12 (8.7)	7 (12.3)	0.443
**Remission Ca, N (%)**	7 (3.7)	0 (0)	0.383	9 (6.5)	0 (0)	0.060
**Active Ca, N (%)**	7 (3.7)	5 (25)	**<0.001**	10 (7.2)	5 (8.6)	0.772
**Liver disease, N (%)**	2 (1.1)	1 (5)	0.157	0 (0)	1 (1.8)	0.292
**CRF, N (%)**	9 (4.7)	3 (15)	0.093	8 (5.8)	11 (19.3)	**0.004**
**COPD, N (%)**	5 (2.6)	1 (5)	0.545	15 (10.9)	5 (8.8)	0.661
**ILD, N (%)**	2 (1.1)	3 (15)	**<0.001**	3 (2.2)	1 (1.8)	1.000
**Asthma, N (%)**	15 (7.9)	0 (0)	0.371	5 (3.6)	1 (1.8)	0.673
**Drug treatment (yes)**	77 (48.1)	9 (64.3)	0.246	95 (81.2)	33 (76.7)	0.533
**Immunosuppression use**	23 (12.2)	8 (40)	**0.003**	13 (9.4)	5 (8.8)	0.898
**ICU transfer**	39 (20.5)	19 (95)	**<0.001**	36 (26.1)	56 (96.6)	**<0.001**
**Length of hospital stay, days**	7 (1–50)	15 (1–36)	**0.005**	9 (2–45)	12 (1–38)	**0.006**

Continuous variables are expressed as means ± SD or medians (minimum–maximum) and categorical variables as numbers with percentages (in parentheses). Values below *p* < 0.05 are shown in bold. CHF: chronic heart failure, CAD: coronary artery disease, HT: hypertension, DM: diabetes mellitus, CVD: cerebrovascular disease, Ca: cancer, CRF: chronic renal failure, COPD: chronic obstructive pulmonary disease, ILD: interstitial lung disease, ICU: intensive care unit.

**Table 2:** Laboratory parameters of the older population and the adult group according to survival status.

Variables	<65 Years	<65 Years		≥65 Years	≥65 Years	
	Survivors (n = 190)	Non-Survivors (n = 20)	*p*	Survivors (n = 139)	Non-Survivors (n = 58)	*p*
**WBC count (mcL)**	6395 (580–27,180)	8695 (2930–54,730)	**0.015**	6680 (1640–18,620)	7835 (2440–34,060)	0.076
**Lymph count (mcL)**	1140 (220–18,620)	935 (440–20,880)	**0.028**	980 (170–7480)	975 (190–23,680)	0.640
**Neu count (mcL)**	4050 (80–24,400)	7345 (920–21,060)	**0.001**	5100 (980–15,980)	6075 (570–17,930	0.155
**Neu/Lymph**	3.32 (0.09–30.89)	8.67 (0.71–29.09)	**0.001**	5.01 (0.69–37.19)	6.13 (0.39–22.94)	0.235
**PLT count (mcL)**	203,000 (49,000–613,000)	202,000 (25,000–415,000)	0.478	219,000 (17,000–929,000)	177,500 (69,000–515,000)	**0.007**
**Urea (mg/dL**	26 (10–144)	45 (24–183)	**<0.001**	39 (18–183)	63.5 (23–319)	**<0.001**
**Crea (mg/dL)**	0.88 (0.31–7.8)	1.12 (0.62–10.77)	**<0.001**	0.93 (0.33–8.97)	1.27 (0.48–8.56)	**<0.001**
**ALB (g/L)**	39.7 (18.2–50.3)	35.85 (21.9–41.9)	**0.001**	35.8 (17.7–45.6)	35.65 (19.3–42.6)	0.753
**CRP (mg/L)**	36.2 (0.31–396.9)	90.78 (30.8–338)	**<0.001**	74.57 (1.18–396.89)	108.6 (8.9–364.4)	0.119
**Procalcitonin (mcg/L)**	0.07 (0.02–5.35)	0.34 (0.08–6.93)	**0.023**	0.115 (0.02–14.38)	0.29 (0.09–19.12)	**0.01**
**Ferritin (mcg/L)**	363.5 (3.75–7371)	1048 (277–10,540)	**<0.001**	362.5 (14.8–2685)	585 (32.9–3498)	**0.039**
**LDH (IU/L)**	249.5 (116–1285)	520 (240–926)	**<0.001**	280 (126–644)	322 (157–1289)	**0.04**
**PNI**	45.65 (21.1–141.4)	40.4 (24.7–136.9)	**0.003**	40.75 (26.15–74.9)	41.62 (25.2–158.7)	0.739
**SII**	646 (16.5–18,935)	1854 (25–5672)	**0.005**	1104 (76.5–9196)	1111 (62–7096)	0.993

Continuous variables are expressed as means ± SD or medians (minimum–maximum) and categorical variables as numbers with percentages (in parentheses). Values below *p* < 0.05 are shown in bold. WBC: white blood cell, Lymph: lymphocyte, Neu: neutrophil, PLT: platelet, CREA: creatinine, ALB: albumin, CRP: C-reactive protein, LDH: Lactate dehydrogenase, PNI: prognostic nutritional index, SII: systemic immune inflammation index.

## Data Availability

The datasets analyzed during the current study are available from the corresponding author on reasonable request. This research was conducted with the permission of the Turkish Health Ministry Scientific Research Committee and Ethics Committee of Ege University Medical Faculty and the data are not publicly available due to privacy and ethical restrictions.

## Supplementary Materials

The following supporting information can be downloaded at: https://www.mdpi.com/article/10.3390/jcm13154466/s1, Table S1 The characteristics of the total group according to survival status. Figure S1. ROC Curve of PNI, and SII in the total group.
