# Supplementary material for: Predictivity of the Prognostic Nutritional Index and Systemic Inflammation Index for All-Cause In-Hospital Mortality in Geriatric and Adult COVID-19 Inpatients
Source: J Clin Med. 2024 Jul 30;13(15):4466. doi: 10.3390/jcm13154466 (PMC11313282; doi:10.3390/jcm13154466)
Supplement: Supplementary file 1 [file jcm-13-04466-s001.zip › jcm-3039470-supplementary.pdf]

Supp. Table-S1 The characteristics of the total group according to survival status

	General (N=407)	Survivors (N=329)	Non-survivors (N=78)	P value
**Age, y**	61.8±15.8	62 (18-91)	74 (34-95)	**0.000**
**Male, N (%)**	238 (58.5)	189 (57.5)	49 (62.8)	0.387
**Female, N (%)**	169 (41.5)	140 (42.5)	29 (37.2)	0.387
**Respiratory rate, min^{-1}**	20.4±2.9	20 (14-37)	22 (16-38)	**0.000**
**Heart rate, bpm**	90.1±16.3	88 (55-149)	88 (60-141)	0.362
**Comorbidity, N (%)**	305 (74.9)	235 (71.4)	70 (89.8)	**0.001**
**CHF, N (%)**	18 (4.4)	13 (4)	5 (6.5)	0.355
**CAD, N (%)**	52 (12.8)	37 (11.3)	15 (19.5)	0.053
**HT, N (%)**	159 (39.1)	119 (36.3)	40 (51.9)	**0.011**
**DM, N (%)**	106 (26.0)	86 (26.2)	20 (26)	0.965
**CVD, N (%)**	20 (4.9)	13 (4)	7 (9.1)	0.077
**Remission Ca, N (%)**	16 (3.9)	16 (4.9)	0 (0)	0.050
**Active Ca, N (%)**	27 (6.6)	17 (5.2)	10 (12.8)	**0.022**
**Liver Disease, N (%)**	4 (1.0)	2 (0.6)	2 (2.6)	0.165
**CRF, N (%)**	31 (7.6)	17 (5.2)	14 (18.2)	**0.000**
**COPD, N (%)**	26 (6.4)	20 (6.1)	6 (7.8)	0.606
**ILD, N (%)**	9 (2.2)	5 (1.5)	4 (5.2)	0.071
**Asthma, N (%)**	21 (5.2)	20 (6.1)	1 (1.3)	0.147
**WBC count (mcL)**	6810 (580-54730)	6610 (580-27180)	8025 (2440-54730)	**0.001**
**Lymph count (mcL)**	1050 (170-23680)	1080 (170-18620)	955 (190-23680)	**0.017**
**Neu count (mcL)**	4810 (80-24400)	4510 (80-24400)	6450 (570-21060)	**0.000**
**Neu/Lymph**	4.4 (0.1-37.2)	3.95 (0.1-37.2)	6.69 (0.39-29.09)	**0.000**
**PLT count (mcL)**	204000 (17000-929000)	209000 (17000-929000)	182000 (25000-515000)	**0.014**
**Urea (mg/dL)**	35 (10-319)	32 (10-183)	61.5 (23-319)	**0.000**

**Crea (mg/dL)**	0.9 (0.3-10.8)	0.9 (0.3-7.8)	1.26 (0.48-10.8)	**0.000**
**ALB (g/L)**	37.8 (17.7-50.3)	38.1 (17.7-50.3)	35.7 (19.3-42.6)	**0.000**
**CRP (mg/L)**	59.2 (0.3-396.9)	53.38 (0.3-396.9)	101.2 (8.9-364.4)	**0.000**
**Procalcitonin (mcg/L)**	0.1 (0.02-19.12)	0.09 (0.02-14.38)	0.29 (0.08-19.12)	**0.000**
**Ferritin (mcg/L)**	429 (3.8-10540.0)	363.5 (3.75-7371)	612 (32.9-10540)	**0.000**
**LDH (IU/L)**	275 (116-1289)	263 (116-1285)	332 (157-1289)	**0.000**
**PNI**	43.5 (21.1-158.7)	43.95 (21.1-141.4)	41.6 (24.7-158.7)	**0.000**
**SII**	875.2 (16.5-18935.6)	816.48 (16.5-18935.6)	1179.31 (25-7096.7)	**0.013**
**Drug Treatment (Yes), N (%)**	214 (52.6)	172 (62.1)	42 (73.7)	0.097
**Immunosupression Use, N (%)**	49 (12.0)	36 (11)	13 (16.9)	0.153
**ICU transmission, N (%)**	150 (36.9)	75 (22.9)	75 (97.4)	**0.000**
**Length of hospital stay, day**	9 (1-50)	8 (1-50)	13 (1-38)	**0.000**

Continuous variables were expressed as means±SD, or medians (minimum-maximum), and categorical variables as numbers with percentages (in parentheses). Values below p<0.05 were shown bold.

CHF; coronary heart failure, CAD; coronary artery disease, HT; hypertension, DM; diabetes mellitus, CVD; cerebrovascular disease, Ca; cancer, CRF; chronic renal failure, COPD; chronic obstructive pulmonary disease, ILD; interstitial lung disease, WBC; white blood cells, Lymph; lymphocyte, Neu: neutrophil, PLT; platelet, CREA;creatinin, ALB;albumin, CRP; C-reactive protein, LDH; Lactate dehydrogenase, PNI; prognostic nutritional index, SII; systemic immune inflammation index, ICU; intensive care unit

Supp. Figure S1. ROC Curve of PNI, and SII in the total group

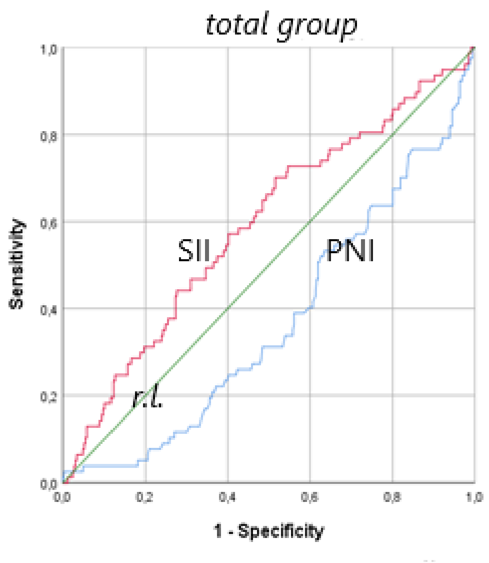

SII; systemic inflammation index, PNI; prognostic nutritional index, r.l.; reference line

In the total population, AUC of PNI was 0.631 (95% CI 0.564-0.698) (P<0.001) and AUC of SII was 0.591 (95% CI 0.519-0.662) (P=0.013).
